# Nursing in America

**Published:** 1887-01-22

**Authors:** Alice Fisher

**Affiliations:** Matron of the Philadelphia Hospital, West Philadelphia


### A Noble Profession: Nursing the Sick.

### NURSING IN AMERICA.

By Miss Alice Fisher,
Matron of the Philadelphia Hospital, West Philadelphia.

I have received so many letters from English nurses desirous of obtaining employment here, that I offer the following remarks for their consideration, always premising that my opinion is founded on my own short experience of two years in America.

No woman should attempt work in this or any foreign country unless she can rely with all human confidence on her good health, and her ability to endure change of climate, of food, and of manner of living. It is also absolutely necessary for her own comfort, and for her work's sake, that she should possess a distinct power of ready adaptation of herself to circumstances. A woman of middle age, who is not a thorough citizen of the world, had better stay at home. Desirable appointments and high wages are to be found in America; but nowhere is so good a sixpennyworth required for sixpence, and people who cannot get on in England are not likely to do so here. The social conditions of life are simpler than in England, and an educated private nurse is very commonly treated as one of the family, except in very rich houses, where much the same arrangements obtain all over the civilised world. In all cases in America a nurse receives the very greatest consideration, though it is possible that she may be required to do some things which would not be expected of her in England. I will give an instance of this: A doctor, the other day, engaged for a confinement the services of one of my nurses who had just graduated. He offered twenty dollars (four pounds) a week, and would have been willing to give five dollars more, if necessary; but he stipulated on the part of the lady that the nurse should do all the baby's washing during the month. She hesitated a moment, but she was a sensible girl, and went. A woman who undertakes private nursing in America should also be able and quite ready to cook little delicacies for her patient. People who have the means to pay for private nurses when they are ill, leave the great cities about June, and do not return till October. I think, therefore, that the latter month would be the best in which to commence private nursing. Philadelphia, Pittsburg, and other large cities would be desirable places to settle in. Boston and New York, by the way, should be avoided, as both, I understand, are already overstocked with nurses. A nurse should bring with her a diploma or certificate of qualification, a few good testimonials from doctors, and one from the Lady Superintendent of the hospital where she was trained. These should be printed and, with her card, left at the houses of the principal medical men. She may also advertise in a local paper. In Philadelphia, at least, there is an admirably conducted registry for nurses, and a competent and attractive nurse who enters her name early in the autumn season, is pretty sure of employment. A great many nursing women in this city live together in communities of six or eight, taking a certain number of rooms in a house, sharing expenses, attending to each other's interests during absence, etc. There are also hospitals to which a private nursing service is attached, but I am not certain whether these receive graduates of other training schools; or, if so, under what conditions. I hope before long, as some interest appears to have been excited in the matter, to collect reliable facts as to the most promising "locations" for English nurses, their chances of success, and the probable preliminary outlay which would be necessary.

